# Neurobiology of Pain

**DOI:** 10.1016/j.ynpai.2016.09.001

**Published:** 2016-11-02

**Authors:** Fernando Cervero

As I start editing a new scientific journal I must give an honest answer to a very simple question: do we really need another journal? Since there are already thousands of journals that cover every imaginable aspect and form of scientific enquiry, what will be unique in *Neurobiology of Pain*?

My answer to this question is twofold. On the one hand I do believe very strongly that we have the duty of providing scientists with high quality vehicles for their reports. The world of scientific publishing has gone through a revolutionary period imposed by new technologies and new business models and it is essential that we offer our colleagues a first class and genuine vehicle for their science. This is the first aim of our new journal: a high quality journal for the best science. To achieve this goal we have the support of Elsevier, the number one scientific publisher in the world, and of a first class editorial board that includes the top pain scientists from around the globe. We do have the instruments to make *Neurobiology of Pain* the vehicle of choice for the best science on the mechanisms of pain,

Which brings me to the second part of my answer to the “why?” question. There is a great deal of scientific interest in the study of pain as a neurological problem and in the development of newer and better pain therapies. This awareness is the consequence of society’s demand for more effective pain relief and of people’s unwillingness to keep considering pain as a test of character that must be endured. An immediate consequence of this societal demand has been the interest of the pharmaceutical industry in better analgesics that has, in turn, driven scientific enquiry into the mechanisms and management of pain.

There are already several very good and well-established pain journals but I believe that the increased interest on the topic shows that there is room for more. And here is where *Neurobiology of Pain* can fill a niche. What will make our journal unique, in addition to our commitment to high quality, fair reviewing and fast publication, is that the journal is focused exclusively on basic science studies of pain mechanisms, covering the full range of topics, from cellular and molecular mechanisms to psychophysical studies of pain processing. And that our new journal will expand the rigid limits of standard research papers to include topical reviews, discussion papers, consensus reports, negative results and technical evaluations. We aim to cover every study, every aspect and every scientific vehicle that will increase our knowledge of the neurobiological mechanisms of pain.

We have made considerable progress in the understanding of pain mechanisms but much more remains to be made. Below I will point out a few areas that are still obscure or that need brand new knowledge. While we will consider for publication papers on every aspect of pain neurobiology, I would particularly welcome studies on those topics of current interest where more solid science is much needed.

Perhaps the most important question in pain science is how an acute noxious stimulus can transform the nervous system to the extent of generating persistent and chronic pain. And why this only happens in a fraction of individuals, despite similar triggering events. These are key questions in pain neurobiology: how and by which mechanism can pain switch from being a protective sensation to becoming a horrific curse. And what makes certain individuals susceptive to pain chronification.

Immediately related to this question is the role of the genetic constitution of the individual to pain perception and pain chronification. Human genetics is progressing in leaps and bounds but we still do not know how much a certain genetic profile determines pain sensitivity. Equally, we are not sure about the relevance of environmental factors in the development of chronic pain or in the differences in pain perception between individuals, genders and populations. Clarifying the role of genetics and the environment in pain perception – nature *vs* nurture – is essential to fully understand the mechanisms of pain.

Another important topic in pain neurobiology is the role of increased sensitivity of nociceptive pathways in the generation of chronic and persistent pain. Much has been made of the rather nebulous concept of “sensitization”, a word that seems to mean different things to different people, and an idea that has been judged, without much hard evidence, to be responsible for every form of pain, particularly of those syndromes whose etiology is obscure. Yet, we are not sure that increased excitability of neurons underlies persistent pain, especially those forms of pain that last for months or years. Not enough attention has been given to structural and functional alterations of the nervous system not necessarily linked to enhanced excitability. Much remains to be sorted out on the topic of sensitization.

The failure of experimental pain models to mimic clinical pain conditions has cast doubts on the usefulness of fundamental research in the search for new analgesics. This is an area where our journal can make a considerable impact. We focus on the neurobiology of pain, and hence on experimental pain studies, but we also aim to understand clinically relevant pain conditions. We do need reassurance as to the usefulness of experimental models and we can only get this guarantee by well-designed and well-executed studies on the relationship between a pain model and a clinical pain disease.

Finally, we are also short of knowledge on the roles of stress and hormonal alterations in the perception of pain. We know that there are pain conditions directly linked to stress, both physical and emotional, that some forms of chronic pain are many times more prevalent in women and that there are comorbidities of chronic pain syndromes with cognitive and psychological impairments. But we still do not know what are the neurobiological mechanisms that link all these various alterations. Good science on these topics is urgently required to remove the stigma of mental disorder that is often attached to chronic pain sufferers.

I hope to have answered satisfactorily the main question that I face as the Editor-in-Chief of our new journal. I am committed to producing a high quality journal specifically devoted to the neurobiology of pain, developed by the most experienced scientific publisher in the world and run by a first-class international board. We are poised, ready and looking forward to receiving your papers.

## Conflict of Interest

There is no conflict of interest.

